# Pattern Recognition Receptors in Multiple Sclerosis and Its Animal Models

**DOI:** 10.3389/fimmu.2019.02644

**Published:** 2019-11-12

**Authors:** M. Elizabeth Deerhake, Debolina D. Biswas, William E. Barclay, Mari L. Shinohara

**Affiliations:** ^1^Department of Immunology, Duke University School of Medicine, Durham, NC, United States; ^2^Department of Molecular Genetics and Microbiology, Duke University School of Medicine, Durham, NC, United States

**Keywords:** pattern recognition receptors (PRRs), multiple sclerosis (MS), experimental autoimmune encephalomyelitis (EAE), Toll-like receptors (TLRs), NOD-like receptors (NLRs), C-type lectin receptors (CLRs), RIG-I like receptors (RLRs)

## Abstract

Pattern recognition receptors (PRRs) coordinate the innate immune response and have a significant role in the development of multiple sclerosis (MS). Accumulating evidence has identified both pathogenic and protective functions of PRR signaling in MS and its animal model, experimental autoimmune encephalomyelitis (EAE). Additionally, evidence for PRR signaling in non-immune cells and PRR responses to host-derived endogenous ligands has also revealed new pathways controlling the development of CNS autoimmunity. Many PRRs remain uncharacterized in MS and EAE, and understanding the distinct triggers and functions of PRR signaling in CNS autoimmunity requires further investigation. In this brief review, we discuss the diverse pathogenic and protective functions of PRRs in MS and EAE, and highlight major avenues for future research.

## Introduction

Multiple sclerosis (MS) is an autoimmune disorder of the central nervous system (CNS) characterized by neuroinflammation, demyelination, and axon damage. Disease development in MS is likely due to a complex interaction of genetic and environmental factors leading to CNS-targeted autoimmunity, involving activation of both the innate and adaptive immune response (1). In particular, the innate immune response is a critical component of disease development in MS and its animal model, experimental autoimmune encephalomyelitis (EAE) but is less well-studied in MS compared to the adaptive immune system (2). The diverse functions of the innate immune response are orchestrated by pattern recognition receptors (PRRs), which sense both microbial-associated molecular patterns (MAMPs) and damage-associated molecular patterns (DAMPs). PRRs are mainly expressed on innate immune cells including macrophages, dendritic cells (DCs), neutrophils, and microglia, but can also be expressed on non-immune CNS-resident cells. Many PRRs show elevated gene expression in MS (3, 4), and genome-wide associate studies (GWAS) have identified variants in multiple PRRs linked to increased MS risk (5–7).

**Figure 1:**
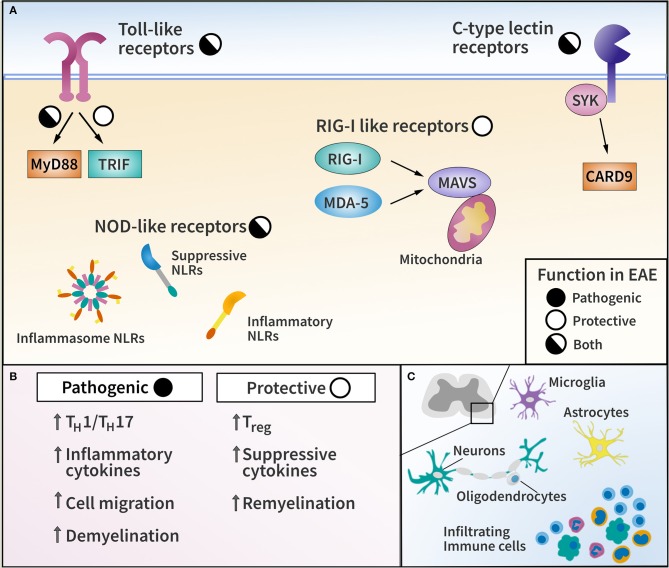
Pattern recognition receptor (PRR) families and their functions in EAE. **(A)** Diagram of major PRR families with pathogenic or protective function in EAE as indicated. **(B)** List of representative effector mechanisms to elicit pathogenic and protective function by PRRs. **(C)** Diagram of CNS-resident cells and CNS-infiltrating immune cells capable of expressing PRRs in EAE and MS.

The major families of PRRs include Toll-like receptors (TLRs), Nod-like receptors (NLRs), C-type lectin receptors (CLRs), and RIG-I like receptors (RLRs). TLRs and CLRs are transmembrane proteins which recognize diverse MAMPs and DAMPs. NLRs and RLRs, on the other hand, are located in the cytoplasm where they function as sensors to modulate the immune response. These PRR families can have both protective and pathogenic functions in EAE, depending on context (Table 1). Here, we summarize the diverse roles of PRR signaling in EAE and MS (Figure 1), and highlight outstanding questions in the field.

**Table 1:** Functions of pattern recognition receptors (PRRs) in the EAE model of multiple sclerosis.

	**PRRs**	**Function**	**Approach**	**References**
TLRs	TLR1	N.D.	*Tlr*1^−/−^ mice	(18)
	TLR2	N.D.	*Tlr2*^−/−^ mice	(19)
		Pathogenic	*Tlr2*^−/−^ mice (recipient, passive EAE)	(22)
		N.D.	*Tlr2*^−/−^ mice (male)	(18)
		Pathogenic	*Tlr2*^−/−^ mice (female)	(18)
		Pathogenic	*Tlr2*^−/−^ mice (female recipient, passive EAE)	(18)
		Pathogenic	*Tlr2*^−/−^ mice	(21)
		Protective	Agonist (Pam2CSK4)	(14)
		Protective	Agonist (L654)	(14)
	TLR3	Protective	Agonist [poly(I:C)]	(35)
	TLR4	N.D.	*Tlr4*^−/−^ mice	(18)
		Protective	*Tlr4*^−/−^ mice	(28)
		Pathogenic	*Tlr4*^−/−^ mice	(24)
		Protective	Agonist (LPS)	(26)
		Protective	Agonist (LPS)	(27)
	TLR6	N.D.	*Tlr6*^−/−^ mice	(28)
	TLR9	Protective	*Tlr9*^−/−^ mice	(28)
		Pathogenic	*Tlr9*^−/−^ mice	(18)
NLRs	NOD1	Pathogenic	*Nod1*^−/−^ mice	(21)
	NOD2	Pathogenic	*Nod2*^−/−^ mice	(21)
	NLRP3	N.D.	*Nlrp3*^−/−^ mice	(58)
		Pathogenic	*Nlrp3*^−/−^ mice	(30)
		Pathogenic	*Nlrp3*^−/−^*Rag2*^−/−^ mice (recipient, passive EAE)	(53)
		Pathogenic	*Nlrp3*^−/−^ mice	(52)
		Pathogenic	*Nlrp3*^−/−^ mice	(31)
		N.D.	*Nlrp3*^−/−^ mice (aggressive EAE)	(31)
		Pathogenic	Inhibitor (MCC950)	(56)
		Pathogenic	Inhibitor (JC-171)	(92)
	NLRC3	Protective	*Nlrc3*^−/−^ mice	(63)
		Protective	*Nlrc3*^−/−^ mice	(62)
	NLRX1	Protective	*Nlrx1*^−/−^ mice	(67)
	NLRP12	Pathogenic	*Nlrp12*^−/−^ mice	(70)
		Protective	*Nlrp12*^−/−^ mice	(71)
		Protective	*Nlrp12*^−/−^ mice	(72)
RLRs	MAVS	Protective	*Mavs^−/^*^−^ mice	(91)
	RIG-I	Protective	Agonist (3pRNA)	(91)
	MDA-5	Protective	Agonist [complexed poly(I:C)]	(91)
CLR	MICL	Pathogenic	*Clec12a*^−/−^ mice	(93)
		Pathogenic	Inhibitor (blocking antibody)	(93)
	DCIR2	Protective	*Clec4a4^−/^*^−^ mice	(88)
	Dectin-1	Protective	Agonist (zymosan)	(84)

*N.D., Not Detected*.

## Toll-Like Receptors (TLRs)

The TLR family includes both surface and endosomal transmembrane receptors, and is comprised of 10 TLRs in human (TLR1–10) and 12 in mouse (TLR1–9, 11–13). TLRs are studied mainly in peripheral myeloid cells, but can also be expressed by non-immune cell types. In the CNS, microglia express a large array of TLRs, while non-immune cells, such as astrocytes and oligodendrocytes have a more limited repertoire (8).

TLR2, in particular, has been implicated by multiple studies in MS pathology. TLR2 is highly upregulated in peripheral blood mononuclear cells (PBMCs), cerebrospinal fluid (CSF) cells, and demyelinating lesions in MS patients (8). Consistent with a pathogenic function, TLR2 stimulation of regulatory T cells (T_reg_) from MS patients reduced T_reg_ suppressive functions and promoted a shift toward an inflammatory T_H_17 response (9). Among non-immune cells, TLR2 is expressed by oligodendrocytes in MS lesions, and TLR2 ligation with hyaluronan inhibits the maturation of oligodendrocyte progenitor cells (OPCs) *in vitro* (10). In addition, relapsing-remitting MS (RRMS) patients show elevated levels of soluble TLR2 (sTLR2) (11), although sTLR2 would not necessarily be pathogenic because it could absorb TLR2 ligands. Paradoxically, significantly lower levels of microbiome-derived TLR2 ligands were found in the blood of MS patients (12, 13). As discussed below, later EAE studies suggest that TLR2 can in fact be protective (14–16) through a mechanism known as TLR tolerance. Beyond TLR2, other receptors including TLR3 and TLR4 are also expressed in active MS lesions (8), and TLR2, TLR4, TLR9 all showed elevated expression on CD4^+^ and CD8^+^ T cells from MS patients (9, 17). Expression of TLRs by T_H_17 cells in particular was shown to correlate with the extent of brain lesions and neurological disabilities in MS (17).

TLR signaling is mediated either by MyD88 or TRIF. Multiple EAE studies have identified distinct functions for TLR signaling through these two different pathways. Specifically, MyD88 is highly pathogenic in EAE, as *Myd88*^−/−^ mice do not develop any disease symptoms (18, 19). Thus, it was expected that TLRs, which signal through MyD88 (TLR1, 2, 4, and 6), would also be pathogenic. However, the role of TLRs in EAE is not so straightforward. Despite similar EAE severity between *Tlr2*^−/−^ and wild-type mice in some reports (19, 20), and other results suggesting the pathogenicity of TLR2 (18, 21, 22), more recent studies indicate TLR2 stimulation may also be protective. In particular, treating mice with low-dose TLR2 ligands reduced EAE severity (14, 16). Interestingly, in the cuprizone model of demyelination, which is not mediated by autoimmunity, TLR2 stimulation also demonstrates a therapeutic benefit by enhancing remyelination (15). This protective effect of TLR2 stimulation is thought to be mediated by TLR tolerance, a process in which repeated stimulation of the receptor leads to a dampened response to subsequent stimuli and a more regulatory response. In contrast, *Tlr2*^−/−^ mice developed reduced pathology in the cuprizone demyelination model, suggesting a pathogenic role for basal TLR2 signaling in the absence of exogenous stimulation (15, 23). In summary, multiple studies suggest that TLR2 signaling may be either pathogenic or protective in EAE, depending on context and timing (14), but further studies are needed to understand the mechanism of how these distinct functions are mediated.

Similar to TLR2, TLR4 is also known to be both pathogenic (24, 25) and protective (18, 26–28) in EAE. Other MyD88-dependent TLRs include endosomal TLR7, TLR8, and TLR9, which induce Type-1 interferons (IFN-I) expression. Again, some reports indicated a pathogenic role of TLR7, TLR8, and TLR9 (18, 19, 29), while others found TLR9 to be protective (28). The discrepancy in TLR9 results may be attributed to different EAE induction methods. The studies suggesting TLR9 to be pathogenic applied a booster immunization on Day 7 (18, 19), but no booster was applied in the latter study, which found TLR9 to be protective (28). EAE induction with a booster immunization is a similar method to what we previously described as “Type-B EAE,” a distinct EAE subtype resistant to IFNβ treatment (30, 31). Therefore, with the strong EAE induction with repeated immunization, IFN-I generated by TLR9 stimulation may not inhibit EAE. Instead, the pathogenicity of TLR9 may be enhanced by TLR9-mediated pro-inflammatory cytokine production. TLR8, identified in the spinal cord axons during EAE (32), was demonstrated to inhibit neurite growth and enhance neuronal apoptosis (33), possibly serving as a pathogenic receptor in EAE. Lastly, TLR3 which is endosomal, transduces its signal through TRIF but not MyD88, and appears to be protective in EAE. *Trif* ^−/−^ mice exhibit severe EAE, likely due to reduced IFN-I expression leading to an enhanced Th17 response (34). Indeed, the TLR3 ligand, poly(I:C), suppressed EAE by elevating the production of IFN-I (35). In summary, some endosomal TLRs (TLR3, 7, 9) appear to be protective in IFNβ-responsive EAE subtypes (30, 31), as these TLRs strongly induce protective IFN-I expression (36).

## NOD-Like Receptors (NLRs)

NLRs are an evolutionarily ancient family of receptors and potent regulators of inflammation and immunity (37, 38). They are intracellular, and regulate danger signals through pre- and post-translational mechanisms (37). Collectively, NLRs possess a diverse array of functions, including NFκB activation/inhibition, gene transcription, and formation of inflammatory signaling platforms termed inflammasomes (37, 38).

Some NLRs are now known to be associated with MS, though most remain uninvestigated. As inflammasome-forming NLRs, NLRP1, NLRP3, and NLRC4 are closely associated with inflammatory immune reactions, and have been linked to MS risk. Specifically, SNPs in the *NLRP1* and *NLRP3* loci have been associated with MS (39–43). Expression levels of *NLRP3* mRNA in PBMCs correlate with disease relapse in RRMS patients (43), and *ex vivo* stimulation of PBMCs from primary progressive MS patients also showed enhanced NLRP3 expression and activation (44). NLRC4 protein is abundantly expressed in MS lesions, and most prominently in lesion-associated astrocytes (45). Loss of function *NLRC4* mutations are also associated with improved response to IFNβ treatment (6). Although NOD2 stimulation on DCs enhances Th17 responses in human T cells *ex vivo* (46), SNPs in neither *NOD1* nor *NOD2* are associated with MS risk (47).

NLRs have both pathologic and protective functions in the EAE model. Both NOD1 and NOD2 are pathogenic in EAE (21), and T cells in *Nod1*^−/^^−^ and *Nod2*^−/^^−^ mice do not accumulate in the CNS, presumably due to poor cell migration or reduced antigen presentation in the CNS (21). NLRP1, an inflammasome-forming NLR, is highly expressed in the CNS, specifically by neurons (48–50), astrocytes (51), and oligodendrocytes (48). In the non-autoimmune cuprizone model of demyelination, the NLRC4 inflammasome is functional in both microglia and astrocytes, and contributes to demyelination (45). The NLRP3 inflammasome detects a wide range of sterile and pathogen-derived insults to trigger its activation. Under some conditions, *Nlrp3*^−/^^−^ mice are resistant to EAE (30, 31, 52, 53), due to their inability to promote immune cell migration to the CNS via IL-1β and IL-18 priming, despite the presence of pathogenic Th17 cells (30). *Nlrp3*^−/^^−^ mice are also protected from cuprizone-induced demyelination (54), in which T cell involvement is minimal (55). These results suggest multiple roles for NLRP3 in CNS inflammation and demyelination. Indeed, while inhibition of NLRP3 with MCC950 potently suppresses traditional EAE (56), it also suppresses mechanical allodynia in a neuropathic pain model of EAE (57). Nevertheless, under specific circumstances, EAE can be induced in *Nlrp3*^−/^^−^ and *Asc*^−/^^−^ mice, which are also inflammasome-incompetent (31, 53, 58). EAE in the absence of the NLRP3 inflammasome exhibits an atypical pathogenesis with brain-targeted inflammation (31). IFNβ, a treatment for RRMS which inhibits NLRP3 inflammasome activation, is ineffective in treating this form of EAE (31, 53), which is instead dependent on CXCR2 and lymphotoxin (31). This relationship between IFNβ and NLRP3 in EAE may reveal insights into the heterogeneity of response to IFNβ treatment within the MS patient population (31).

In contrast to the inflammation-inducing NLRs, NLRC3, NLRX1, and NLRP12 are protective in EAE. NLRC3 negatively regulates inflammatory pathways in innate and adaptive immune cells (59–61) by suppressing NFκB activation (61) and IFNβ release (60). *Nlrc3*^−/^^−^ mice demonstrate more severe EAE (62, 63) with enhanced DC priming of pathogenic T cells (62). NLRX1 localizes to the mitochondrial outer membrane (64) and interferes with MAVS signaling and induction of IFN-I (64, 65). NLRX1 suppresses NFκB induced by TLR signaling (65, 66) and *Nlrx1*^−/^^−^ mice are more susceptible to EAE (67) due to hyperactivation of myeloid cells in the CNS (67). Notably, both NLRC3 and NLRX1 are protective despite having the potential to suppress IFN-I induction. NLRP12 also suppresses NFκB activation (68, 69), and the lack of *Nlrp12* results in atypical EAE, with ataxia and balance deficits (70). These *Nlrp12*^−/^^−^ mice had increased infiltration of T cells into the brain rather than the spinal cord, as well as an increase in Th2 cells over Th1 and Th17 subsets (70). Another report demonstrated that *Nlrp12*^−/^^−^ mice develop more severe, but traditional EAE (71, 72), however, both studies agreed upon a T cell-intrinsic suppressive function of NLRP12 (70–72). The reason for these different EAE phenotypes is unclear, though the *Nlrp12*^−/^^−^ mice used in these studies were generated independently with different deletions in exon 2 (70) or exon 3 (71, 72).

## C-type Lectin Receptors (CLRs)

CLRs are a large family of carbohydrate-recognition domain (CRD) containing proteins, a subset of which have described immune functions. CLRs can recognize both MAMPs and DAMPs, and mediate their response by multiple pathways. Most commonly, CLRs function through association with immunoreceptor tyrosine-based activation motif (ITAM) or inhibition motif (ITIM) signaling, depending on the receptor (73). Notably, SNPs in the C-type lectin *CLEC16A* gene have been significantly linked to the risk of developing MS in multiple GWAS studies (7, 74–77). Yet, unlike other CLRs, CLEC16A is localized to the cytosol and its function remains relatively uncharacterized (78). Although CLRs have significant and diverse immune functions, their involvement in MS is poorly understood, and only a limited number of studies on CLRs in EAE have been published.

Initial studies of CLR function focused on the ability of some CLRs to recognize and respond to adjuvant used for EAE induction. Specifically, Mincle, MCL, and Dectin-2 recognize components of heat-killed *Mycobacteria* (hk*Mtb*), the most common adjuvant used in complete Freund's adjuvant (CFA) to induce EAE (79–81). These CLRs appear to mediate their response to hk*Mtb* through Syk/CARD9 signaling which can induce pro-inflammatory cytokine production and inflammasome activation, thus promoting pathogenic Th17 differentiation (79, 80). Specific agonists for MCL and Dectin-2 (TDM and Man-LAM, respectively) are sufficient adjuvants to induce EAE (81, 82). In contrast, the TLR2/Dectin-1 agonist zymosan can only induce limited disease compared to hk*Mtb*, while the Dectin-1 specific agonist, curdlan is unable to induce EAE at all (83).

Additional studies suggest that CLR signaling outside the context of adjuvant recognition may be able to limit EAE development. Specifically, the Dectin-1/TLR2 agonist zymosan can ameliorate EAE when administered in either the MOG_35−55_ (B6 mice) or PLP_139−151_ (SJL mice) models of EAE (84), suggesting a potential protective role for Dectin-1 signaling in EAE. Notably, two out of three publications studying CLR function in experimental autoimmune uveitis (EAU) reported data which indicates that Dectin-1-deficient mice develop more severe disease than WT controls (79, 85). However, the role of Dectin-1 in EAU is not clear, and a separate study identified a contradictory pathogenic function for Dectin-1 in EAU (86). The origin of this discrepancy is not known, but may be due in part to experimental approaches which may vary in adjuvant usage or clinical evaluation. Further work is needed to understand the function and mechanism of Dectin-1 in EAE and EAU. Another CLR, DC-SIGN, was reported to recognize endogenous N-glycan modifications of human MOG protein (87). In response to this endogenous ligand, DC-SIGN facilitated phagocytosis of myelin and enhanced IL-10 production by DCs after LPS stimulation in culture. DCIR2 (gene name *Clec4a4*) was also found to regulate DC function and reduce EAE by suppressing TLR-mediated activation and limiting the pathogenic T-cell response (88). Further study is needed to characterize CLR signaling beyond adjuvant recognition, particularly the mechanisms by which some CLRs may be protective while others are pathogenic in the setting of CNS autoimmunity.

## RIG-I Like Receptors (RLRs)

RLRs are cytoplasmic RNA sensors which signal through MAVS to promote IFN-I response during viral infections and in autoimmunity (89). RIG-I and MDA5 are the most well-studied RLRs and are encoded by *DDX58* and *IFIH1* genes, respectively. In the context of MS, one study suggests that combinations of multiple SNPs in genes encoding RLRs (*DDX58, IFIH1, LGP2*) may increase disease risk, although no individual SNPs alone were found to be significantly associated with MS risk (90). In addition, elevated expression of *DDX58* and *IFIH1* was identified in a subset of MS patients with high expression of *MX1*, a marker of active IFN-I stimulation (4).

In EAE, RLR signaling limits disease development (91). Specifically, genetic deletion of *Mavs* exacerbates EAE severity, while administration of RLR ligands [5′-triphosphate dsRNA and poly(I:C)] ameliorates disease in an IFN-I-mediated manner (91). However, as previously mentioned, poly(I:C) can also function as a TLR3 ligand. A better understanding of RLR signaling during EAE and MS could facilitate novel RLR-targeted therapeutic approaches. Since IFN-I production is not the only outcome of RLR stimulation, targeting RLRs might have additional benefits in treating MS in addition to the production of IFN-I.

## Conclusion

Pattern recognition receptors (PRRs) orchestrate the innate immune response in MS and EAE. The pathogenic functions of PRR signaling in CNS autoimmunity are well-described and include promoting pro-inflammatory cytokine production, antigen presentation, and regulation of cell death. In contrast, the protective functions of innate immunity in CNS autoimmunity are less appreciated, yet PRR signaling can regulate the adaptive immune response, restrain innate immune activation, and promote tissue repair pathways. Both MAMPs and DAMPs trigger PRR signaling, but the specific ligands that mediate PRR function in CNS autoimmunity remain poorly characterized. Furthermore, non-immune cells in the CNS including astrocytes, oligodendrocytes, and neurons can also express some PRRs. To understand the role of PRRs in MS and EAE, we will need to better understand detailed mechanisms of the protective functions of PRRs, PRRs ligands in sterile inflammation, and how PRRs in CNS-resident cells modulate disease outcomes.

## Author Contributions

MD, DB, and WB drafted the manuscript under the guidance of MS. MD and DB created the figure and table, respectively. MD, DB, WB, and MS edited and revised drafts. MD, DB, and MS finalized the manuscript. All the authors approved the final version.

### Conflict of Interest

The authors declare that the research was conducted in the absence of any commercial or financial relationships that could be construed as a potential conflict of interest.
